# Associations of U.S. state-level COVID-19 policies intensity with cannabis sharing behaviors in 2020

**DOI:** 10.21203/rs.3.rs-3211086/v1

**Published:** 2023-08-02

**Authors:** Ryan D. Assaf, Rita Hamad, Marjan Javanbakht, Onyebuchi A. Arah, Steven J. Shoptaw, Ziva D. Cooper, Pamina M. Gorbach

**Affiliations:** Benioff Homelessness and Housing Initiative, Center for Vulnerable Populations, Department of Medicine, University of California, San Francisco; Social Policies for Health Equity Research (SPHERE) Program, Department of Social and Behavioral Sciences, Harvard School of Public Health; Department of Epidemiology, Fielding School of Public Health, University of California, Los Angeles; Department of Epidemiology, Fielding School of Public Health, University of California, Los Angeles; Family Medicine and Psychiatry and Behavioral Sciences, David Geffen School of Medicine, University of California, Los Angeles; Department of Psychiatry and Biobehavioral Sciences, University of California, Los Angeles; Department of Epidemiology, Fielding School of Public Health, University of California, Los Angeles

**Keywords:** Cannabis, Policy Evaluation, COVID-19, Respiratory, Pandemic, Public Health, Harm reduction

## Abstract

**Background:**

Cannabis use before the COVID-19 pandemic for many involved sharing prepared cannabis for inhalation, practices that were less prevalent during the pandemic. State-level COVID-19 containment policies may have influenced this decrease. This study examined the extent to which the intensity of state-level COVID-19 policies were associated with individual-level cannabis sharing. Findings have the potential to guide harm reduction policies for future respiratory pandemics and seasonal respiratory virus waves.

**Methods:**

This study used cross-sectional individual-level data from the COVID-19 Cannabis Study, an anonymous U.S.-based web survey on cannabis use disseminated during the early phase of the pandemic (Full sample N = 1,883). We combined individual-level data with state-level policy data from Kaiser Family Foundation’s State COVID-19 Data and Policy Actions for three time-points from June to August 2020 that overlapped with the survey period. Cannabis sharing was dichotomized as any versus no sharing. We adapted a previously published coding framework to score the intensity of COVID-19 policies implemented in each U.S. state and averaged the policy score across the time period. We then used logistic regression models to quantify the associations of the average state-level COVID-19 policy score with cannabis sharing during the pandemic.

**Results:**

Participants (n = 975) reporting using inhalation as a mode for cannabis use were included in this analysis. Most respondents were male (64.1%), non-Hispanic White (54.3%), with a mean age of 33.7 years (SD 8.8). A large proportion (75.1%) reported sharing cannabis during the pandemic. Those who shared cannabis more commonly lived in states with a lower average policy score (15.3, IQR 11.3–19.0) compared to those who did not share (16.3, IQR 13.7–22.7). In adjusted models, the odds of any cannabis sharing per every 5-unit increase in the average COVID-19 policy score were 0.78 (95% CI 0.58, 1.04).

**Conclusions:**

Fewer individuals shared cannabis in states with more intense COVID-19 containment policies compared to those in states with less intense policies. Individuals who use cannabis may be willing to make changes to their behavior and may further benefit from specific and directed public health messaging to avoid sharing during respiratory infection outbreaks.

## Introduction

Cannabis use in the United States is prevalent, with 18.7% of the population (age ≥ 12) reporting past-year use in 2021 (1). Inhalation of cannabis is the most common reported mode of use, including smoked cigarettes (joint/blunts), pipes, water pipes (bongs), cannabis vaporizers (devices that heat cannabis), e-cigarettes (vapes), and rigs (wax/dabs), a pattern that remained during the coronavirus disease (COVID-19) pandemic (2–4). Moreover, cannabis social practices before the pandemic involved using and/or sharing inhaled cannabis (having more than one person put the same device or products in their mouth to inhale) with friends and sometimes with strangers (5–11). We define sharing of cannabis as having more than one person put the same device or products in their mouth to inhale prepared cannabis (e.g., cannabis joints/cigarettes) or cannabis-related paraphernalia (e.g., pipes/rigs/vaporizers).

However, sharing behaviors of paraphernalia for cannabis and other substances are a risk factor for respiratory infections (7–17). This risk of infection may also be true for SARS-CoV-2, the virus that causes COVID-19, through droplets and airborne transmission (12–16). Thus, avoiding sharing of cannabis for inhalation during the pandemic serves as an example of a risk mitigation behavior as it reduces direct exposure to others’ oral fluids that may transmit COVID-19.

Prior work showed that sharing of cannabis shifted for some from higher levels of sharing (always sharing, sharing most of the time) to lower levels (never sharing) during the COVID-19 pandemic compared to before (17). It is important to assess what factors may influence sharing behaviors, specifically for those who reported never sharing during the pandemic, to better tailor harm reduction and public health strategies during future respiratory pandemics and seasonal respiratory waves. One such factor is policies implemented during the COVID-19 pandemic aimed at decreasing or limiting person-to-person contact, e.g., limitations on mass gatherings, stay-at-home orders, closure of non-essential workspaces and schools, and face covering guidance (14, 18). While the U.S. Centers for Disease Control and Prevention (CDC) recommended COVID-19-related policies, policies were ultimately implemented by state and local officials based on conditions relevant to that jurisdiction allowing for variation between states in policy intensity, timing, and duration (19–22).

This study aimed to quantify the magnitude of associations of state-level COVID-19 policy intensity with individual-level sharing of cannabis. We hypothesized that fewer individuals in states with more intense COVID-19 policies reported sharing compared to those in states with less intense policies. Although this study focuses on the COVID-19 pandemic, findings have the potential to guide harm reduction policies for future respiratory pandemics and seasonal respiratory virus waves.

## Methods

### Data

This study used data from the cross-sectional COVID-19 Cannabis Study, an anonymous U.S.-based web survey on cannabis and cannabidiol (CBD) related behaviors disseminated from August 2020 to September 2020. Detailed methods for this survey have been previously described (4). Briefly, survey respondents included in the full study were 18 years of age or older, reported non-medical cannabis, cannabis for medical use, and/or CBD use in the last 12 months, and resided in the U.S. (n = 1,883). Respondents were recruited through online forums (e.g., Reddit, Bluelight, Craigslist, and Twitter), received $5 USD for their participation, and were prevented from “ballot stuffing” by limiting participation to a unique internet protocol (IP) address. In this single survey, participants were asked to recall their non-medical cannabis use behaviors at two 3-month time points: before the COVID-19 pandemic (January to mid-March 2020) and during an early phase of the COVID-19 pandemic (prior 3 months at the time of the survey, June to August 2020; referred to as during the pandemic for the remainder of the paper). Data from this survey include non-medical cannabis frequency of use, mode of use, sharing of cannabis, and demographics (age, sex, education, race/ethnicity, sexual orientation, and state residency). Only respondents who reported a mode of inhalation received questions on sharing behaviors in the survey. Thus, we restricted this study to respondents who reported non-medical cannabis use and self-reported a mode of inhalation for cannabis use in the following ways: smoking (joint/blunt/bong/pipe), vaporizing plant, wax/dab, or vaping oil/concentrates (n = 975).

We then drew state-level exposure and covariate data from three different sources. The first data source was from the Kaiser Family Foundation’s (KFF) State COVID-19 Data and Policy Actions accessed through GitHub repositories (23). Specifically, we used information from 3 time-points (June 4, July 10, August 8, 2020) that overlapped with the survey’s study period “during the pandemic” (June – August 2020). We used dates similarly spaced across the months that could capture variations in policy changes across the period. Data from June 2020 included the following policies: stay-at-home orders; non-essential business closures; larger gathering ban; and restaurant limits. Data from July and August 2020 included all the policies from June, plus the following policies: bar closures and face covering requirements.

The second data source for state-level data was the Johns Hopkins University and Medicine COVID-19 Dashboard by the Center for Systems Science and Engineering with data stored in a GitHub repository from April 4, 2020, until January 12, 2022 (24). COVID-19 data included confirmed infections, deaths, recovered infections, active infections, testing, and hospitalizations by state. For this study, we used the state-level prevalence of confirmed COVID-19 infections from May 24, 2020.

The final data source was from the U.S. Census, which included state population size in 2020, state age distributions in 2020, and state percent urbanicity in 2010 (25, 26). At the time of the analysis, the Census did not have state percent urbanicity beyond 2010.

### Ethics

This study received institutional review board approval from the University of California, Los Angeles (#20-001164). All respondents provided online informed consent.

### Variable Coding and Definitions

The outcome of interest was respondents’ self-reported sharing of cannabis during the COVID-19 pandemic. Respondents used a Likert-scale for agreement with the following question, “I shared joints, blunts, bongs, pipes, vaporizers, or vape pens used for cannabis (marijuana),” with answer choices being never, sometimes, about half the time, most of the time, and always. Because we do not know who was sharing with whom, we dichotomized sharing of cannabis to no sharing (never shared) and any sharing (sometimes, about half the time, most of the time, and always shared).

The exposure of interest was the intensity of state-level COVID-19 policy actions. We scored policies by intensity by adapting a proposed coding framework ranging from 0 to 5, as suggested by Lane et al., and the CDC’s recommended stay-at-home orders (19, 27). In short, a policy scored 5 if the mandate was very high (i.e., all actions prohibited) and 0 for no recommendations or rules implemented for that policy (27). The KFF State COVID-19 Data and Policy Actions data source had policy information on six policies for each U.S. state. These policies included stay-at-home orders, non-essential business closures, bans of large gatherings, restaurant limits, bar closures, and face-covering requirements. For instance, stay-at-home orders included statewide orders, new stay-at-home orders, high-risk groups, rolled back to high-risk groups, lifted, and no state orders. We coded ‘statewide orders’ and ‘new stay-at-home orders’ as 5, ‘high-risk groups’ and ‘rolled back to high-risk groups’ as 4, and ‘lifted’ or ‘no state order’ as 0. Detailed coding of state policies can be found in **Table Supplement 1**. For each month, we summed the values for each of the four specified policies for June (maximum score of 20) and the six specified policies for July and August (maximum score of 30). We then calculated the average policy score for June to August giving a maximum average policy score of 27.

### Covariates

**Figure 1:**
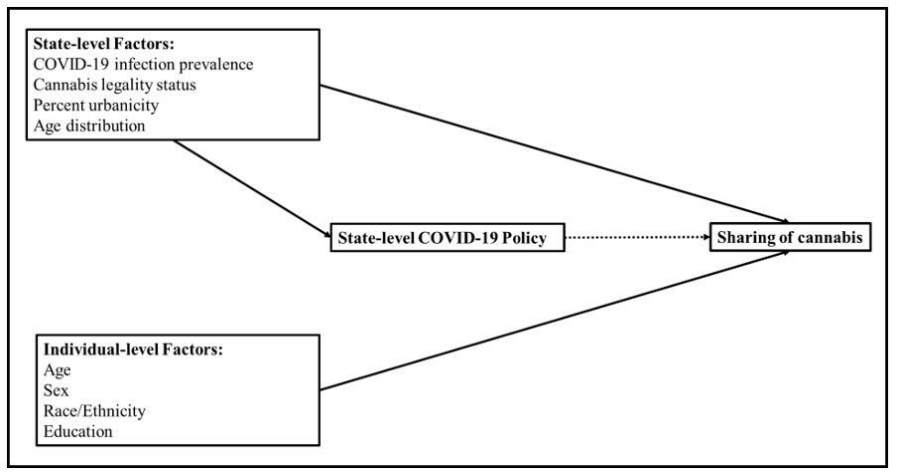
A directed acyclic graph is presented to demonstrate confounders at the population and individual level that may affect the exposure (state’s COVID-19 policy) and the outcome (individual level sharing of cannabis) of interest. These variables included state-level cannabis legality status, state-level COVID-19 infection prevalence, state-level percent urbanicity, state-level age distribution, and individual-level age, sex, race/ethnicity, and education. Measured variables are indicated with a solid box around the variable. Solid arrows depict the causal pathway and dotted arrows depict the measure of interest.

Models were adjusted for potential confounders, encompassing population-level and individual-level variables that may affect the exposure (state COVID-19 policies) and the outcome (individual-level sharing of cannabis). Figure 1 graphically demonstrates these relationships in a directed acyclic graph. These variables included state-level cannabis legality status (as of 2020), COVID-19 infection prevalence, percent urbanicity, and age distribution; and individual-level age, sex, self-reported race/ethnicity, and education of participants. State-level cannabis legality status was categorized as legal for adult use (non-medical use), legal for medical use only, and illegal for medical and adult use (CBD only or fully illegal) (28). State-level COVID-19 infection prevalence was drawn from May 24, 2020, as this precedes the dates used for state’s COVID-19 policy and the outcome (June to August) to minimize issues of temporality. Prevalence was calculated per 100,000 persons given the population size of that state. Finally, individual-level variables, respondent’s age, sex, race/ethnicity, and education, were used to capture variation in the outcome (6–9). Age was recentered at the mean and rescaled per 10-year increases. Race/ethnicity was used as a proxy control for experiences of racism and social, economic, and structural disparities between groups (29, 30). We categorized race/ethnicity as Hispanic/Latinx, non-Hispanic White, non-Hispanic Black, and non-Hispanic other (American Indian/Alaska Native, Native Hawaiian/Pacific Islander, Asian, two or more races, and another race not listed). The latter is a heterogeneous group but was collapsed because of small sample size and unstable estimates. Education was dichotomized as high school/less than high school or greater than high school.

### Statistical Analyses

We calculated frequency distributions, mean with standard deviation (SD), and median with interquartile range (IQR) for demographic variables, policy score, state-level factors, and cannabis behaviors overall and by sharing (no sharing versus any sharing).

We then conducted unadjusted and adjusted logistic regression models to examine the association of the average state-level COVID-19 policy score from June to August 2020 with cannabis sharing during the COVID-19 pandemic. Model 1 was an unadjusted analysis of the average state-level COVID-19 policy association with sharing of cannabis. Model 2 was adjusted for state-level factors: cannabis legality status, COVID-19 infection prevalence, percent urbanicity, and age distribution. Model 3 was adjusted for state-level factors and individual-level variables (age, sex, race/ethnicity, and education). Last, model 4 was adjusted for state-level factors and individual-level variables and included robust standard errors for clustered state data to account for correlated observations within each state (31). As a sensitivity analysis, we ran these models for state-level policy scores in June, July, and August separately to assess variation across the study period. For all models, we rescaled the primary predictor variable so that coefficients would represent the change per 5-unit increase in the COVID-19 policy score.

Overall, missing data were minimal in our study. Data on sharing of cannabis during the pandemic was missing for 0.4% (n = 4) of our sample. Missing data for age (n = 10, 1.0%), sex (n = 12, 1.2%), race/ethnicity (n = 23, 2.4%), and education (n = 13, 1.3%) were minimal, with no missing data on non-medical cannabis use during the pandemic. Thus, we conducted complete case analyses.

All analyses were performed using SAS software Version 9.4 of the SAS System for Windows (SAS Institute Inc., Cary, NC, USA). Mapping of the average COVID-19 policy intensity score by state was performed using R Statistical Software (v4.1.2; R Core Team 2021).

## Results

### Sample Characteristics

Most participants in the overall sample were male (64.1%) and non-Hispanic White (54.3%), with a mean age of 33.7 years (SD 8.8). Among respondents reporting a mode of inhalation for non-medical cannabis use, 810 (83.1%) reported sharing before the pandemic and 732 (75.1%) reported sharing of cannabis during the pandemic. Those who reported sharing during the pandemic were younger than those who did not share, with a mean age of 32.5 (SD 7.9) compared to 35.5 (SD 12.2), respectively. Of those reporting any sharing during the pandemic, 66.1% were male, 33.3% were from the West, and 78.0% reported greater than high school education. On the other hand, 61.7% of those reporting no sharing were male, 52.3% were from the West, and 72.0% reported greater than high school education (Table 1).

### State Characteristics and COVID-19 Policy Score

**Figure 2:**
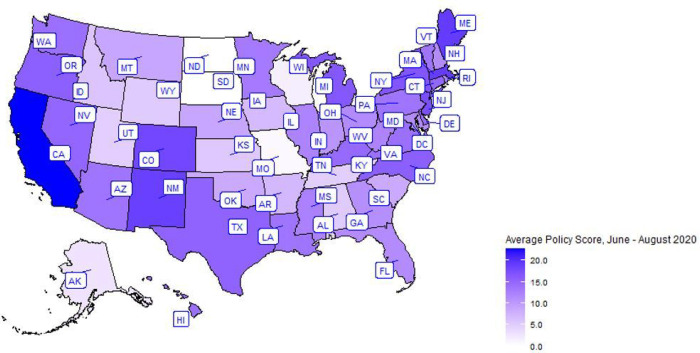
The average COVID-19 policy score from June – August 2020 is presented for each US state. The data source for policy scores were drawn from the was the Kaiser Family Foundation’s (KFF) State COVID-19 Data and Policy Actions accessed through GitHub repositories with policy data by US states starting June 4, 2020, through November 19, 2021. Specifically, we used information from 3 time-points that overlapped with our study period (June – August 2020). The maximum average policy score is 27. A darker shade indicates a higher policy score (more intense policy).

Every U.S. state including the District of Columbia was represented in this study except for Wyoming. Figures 2 shows the average COVID-19 policy score from June to August 2020 by state. Overall, the median of the average policy score was 15.3 (IQR 11.3–19.0). The median policy scores for June, July, and August were 11 (IQR 8–15), 18 (IQR 13–22) and 18 (IQR 14–21), respectively. More of those who reported sharing cannabis lived in states with a lower average policy score (15.3, IQR 11.3–19.0) compared to those who did not share (16.3, IQR 13.7–22.7). Moreover, 45.4% of individuals who reported sharing lived in states with policies for legalized adult use (non-medical / recreational) cannabis laws compared to 60.1% of those who did not share (Table 2–3). Details on the average COVID-19 policy score for June to August 2020, the policy score for each month separately, state-level percent urbanicity, state-level age distribution, state-level cannabis legality, and state-level COVID-19 infection prevalence by state are shown in **Table Supplement 2.**

### Primary Analysis

In the unadjusted model (Model 1), the odds of any cannabis sharing per every 5-unit increase in the average COVID-19 policy score were 0.72 (95% confidence interval [CI] 0.63, 0.83). After adjusting for state-level factors, the odds of any cannabis sharing per 5-unit increase in the average COVID-19 policy score were 0.75 (95% CI 0.61, 0.93) (Model 2). After adjusting for both state-level and individual-level factors, the odds of any cannabis sharing per 5-unit increase were 0.78 (95% CI 0.62, 0.98) (Model 3). Finally, after adjusting for covariates and after accounting for robust standard errors (Model 4), the odds of any cannabis sharing per every 5-unit increase in the average COVID-19 policy score were 0.78 (95% CI 0.58, 1.04) (Table 4).

### Sensitivity Analysis

Results were similar when assessing the COVID-19 policy score for each month (June, July, and August) separately. In Model 4, the odds of any cannabis sharing per every 5-unit increase in June policy score were 0.78 (95% CI 0.58, 1.04), 0.82 (95% CI 0.63, 1.07) per 5-unit increase in July policy score, and 0.82 (95% CI 0.66, 1.02) per 5-unit increase in August policy score (Table 4).

## Discussion

Overall, our study found that individuals in states with more intense COVID-19 policies had lower odds of sharing cannabis compared to those in states with less intense policies. This relationship remained after adjustment for state-level and individual-level covariates. Although, confidence intervals crossed the null when clustering robust standard errors, perhaps due to the loss of effective sample size, most of the interval remained negative in favor of a protective association for cannabis sharing. These findings were consistent for COVID-19 policies in June, July, and August despite changes in policies across these three time-points. Previous studies have assessed state-level COVID-19 policies, masking, and stay-at-home orders on population-level behaviors and found decreases in population movement and COVID-19 infection rates by policy stringency (19–21, 27, 32, 33).

However, few studies have assessed the association of COVID-19 policy on substance use and related behaviors. Most studies that aimed to assess the association of COVID-19 policy, such as stay-at-home orders, did so by comparing waves before and during lockdowns or assessing individual behaviors during lockdowns (34–36). For instance, one longitudinal study assessed tobacco use among young adults during COVID-19 stay-at-home orders compared to before and found reductions in use (34). One challenge in these studies is separating the effect of the pandemic (infection/transmission, socioeconomic consequences, etc.) compared to the effect of COVID-19-related policy. Another challenge is accounting for the variation in intensity, duration, and correlation of implemented COVID-19 containment and closure policies between U.S. states and U.S. counties (22). In order to control for these factors, we aimed to assess the impacts of the intensity of a collection of COVID-19 containment policies and the variations between U.S. states on individuals’ behaviors while accounting for the prevalence of COVID-19 infection at the time.

We found that reported sharing of cannabis was lower among individuals living in states with more intense COVID-19 policies compared to those in states with less intense COVID-19 policy. There may be many mechanisms that could explain this association. First, cannabis use can be a social behavior for some and involves sharing cannabis with others (5–11). COVID-19 policy, such as stay-at-home orders, closure of bars, and large gathering bans, may limit social opportunity for others to connect with friends or strangers. Enforced closure of these public spaces and individual adherence to stay-at-home orders changes where people may have spent their time and reduces one’s opportunity to share cannabis with others, a similar conclusion noted in tobacco studies during the pandemic (34, 37). Second, messaging and policies for COVID-19 may drive perception and emotion (i.e., fear, worry) about COVID-19 infection (38, 39). In turn, these perceptions may influence sharing behaviors (37). Third, there may be unmeasured confounding that was not accounted for or fully controlled (residual confounding) in our study which may partially explain differences in sharing by states with more intense COVID-19 policy compared to those with less intense policy. For instance, there may be a difference in access to cannabis given cannabis legality laws across U.S. states that may allow for those in states with fully legalized cannabis to obtain their own supply compared to those in states without a legalized market. Although we controlled for cannabis legalization by state, there may still be residual confounding that is not accounted for.

Findings from this study may be important during future spikes in COVID-19, during influenza and other respiratory virus seasonal waves, and during future viral respiratory pandemics. This study identified an association with COVID-19 state policy on a behavior that was not the primary aim of policies nor public health messaging. This shows the potential unintended effects or “side effects” that policies may have on other health behaviors such as cannabis sharing. Sharing of paraphernalia for cannabis, tobacco, and crack cocaine inhalation have been shown to be risk factors for respiratory viral and bacterial infections (40–50). Therefore, strategies to reduce or limit sharing during a pandemic of a respiratory illness are important to identify. In the early stage of the pandemic (March – September 2020), messaging with tag lines such as “Puff, Puff, Don’t Pass” and alternatives to sharing cannabis were proposed in newspaper articles, social media community threads, and by national grassroots organizations (5, 11, 51, 52). Additionally, messaging from the World Health Organization and those in tobacco research noted the risk of sharing tobacco products during the pandemic with recommendation to not share (47–50). As cannabis use continues to increase in the United States, tailored public health and harm reduction messaging (i.e., “Puff, Puff, Don’t Pass”) may be important to implement during respiratory viral peaks (51). Interventions for promoting harm reduction messaging for cannabis sharing may include state or county public health departments working with national cannabis grassroots organizations and regulated cannabis dispensaries to provide educational material (17).

### Limitations

There are several limitations to this study. First, the data come from a non-representative convenience sample of highly educated, primarily White male individuals who reported cannabis use and therefore may not be generalizable to all those reporting cannabis use in the U.S. However, this is the only sample, to our knowledge, that assessed cannabis sharing during the pandemic. Second, we do not know with whom sharing was occurring and cannot make conclusions on how this impacted COVID-19 risk. Sharing between intimate partners or household members likely held different risks because of ongoing high levels of exposure whereas sharing with non-household members and people with whom there is no other physical intimacy represents a broader pattern of risk behavior that we were not able to capture. Third, there may be differences in policy actions within states (i.e., at the level of counties/cities) that are not captured here because of data availability on policies in these specific counties at the time of the study (22). Fourth, this was a cross-sectional study. We did not have repeated measures on individuals and state-level policy. We could not control for fixed effects in our model nor conduct a true quasi-experimental analysis like difference-in-differences (pre/post analyses, interrupted time series analyses, etc.). At most, we used robust standard errors for clustered state data to account for correlated observations within each state. Finally, we only looked at one time-point early on during the COVID-19 pandemic (June – August 2020), limiting the generalizability of findings to other periods of the pandemic.

## Conclusion

We found that sharing of cannabis was associated with more intense state-level COVID-19 policies; fewer individuals in states with more intense policies reported sharing compared to those in states with less intense policies. These findings highlight that sharing behaviors may have changed even though COVID-19 policy/messaging was not directed at this behavior. Individuals who use cannabis may be willing to make changes to their behavior and may further benefit from specific and directed messaging to not share during peaks of respiratory infections. There exists a space for collaboration as national grassroots organizations and social media threads proposed messaging to not share early during the pandemic. Future public health messaging should consider harm reduction strategies for cannabis, especially as use continues to increase in the United States.

## Figures and Tables

**Table 1:** Frequency distribution of demographics overall and by cannabis sharing among a national sample of those reporting non-medical cannabis use, August 2020 – September 2020

	Overall	No Sharing	Any Sharing
	n (%)	n (%) ^b^	n (%) ^b^
Total	1883 (100.0)	243 (24.92)	732 (75.08)
**Demographics**			
Age, years (mean [SD])	33.71 (8.77)	35.51 (12.18)	32.52 (7.89)
Sex			
Female	666 (35.92)	90 (37.50)	238 (32.74)
Male	1188 (64.08)	150 (62.50)	489 (67.26)
Sexual orientation ^c^			
LGBQ	576 (31.22)	57 (23.85)	237 (32.78)
Heterosexual	1269 (68.78)	182 (76.15)	486 (32.78)
Race/Ethnicity			
Hispanic/Latino	511 (27.79)	48 (20.87)	238 (32.78)
NH Asian	36 (1.96)	7 (3.04)	15 (2.07)
NH Black	202 (10.98)	30 (13.04)	55 (7.58)
NH American Indian or Alaska Native	51 (2.77)	2 (0.87)	12 (1.65)
NH Native Hawaiian or Pacific Islander	17 (0.92)	1 (0.43)	4 (0.55)
NH White	998 (54.27)	135 (58.70)	393 (54.13)
NH Other ^c^	24 (1.31)	7 (3.04)	9 (1.24)
Education			
Less than high school	160 (8.64)	15 (6.28)	76 (10.45)
High school	215 (11.62)	49 (20.50)	80 (11.00)
Some college credit, no degree	357 (19.29)	57 (23.85)	137 (18.84)
Associates degree	530 (28.63)	33 (13.81)	210 (28.69)
College graduate or higher	589 (31.82)	85 (35.56)	224 (30.81)
Region			
West	709 (37.65)	127 (52.26)	244 (33.33)
Midwest	221 (11.74)	28 (11.52)	90 (12.30)
Northeast	335 (17.79)	42 (17.28)	147 (20.08)
South	618 (32.82)	46 (18.93)	251 (34.29)

Abbreviations. LGBQ= Lesbian, Gay, Bisexual, and Queer; NH= non-Hispanic

a Sharing of cannabis paraphernalia is a sub-question asked only to those who reported using a mode of inhalation based on a check all that apply for smoking (joint/blunt/bong/pipe), vaporizing plant, vaping oil/concentrates, or wax/dab

b May not add to 100% because of missing data

c Non-Hispanic Other= those who reported other race or two or more races

**Table 2:** Distributions of cannabis behavior by cannabis sharing among a national sample of those reporting cannabis use, August 2020 – September 2020

	No Sharing	Any Sharing
	n (%) ^a^	n (%) ^a^
Total	243 (100.0)	732 (100.0)
**Sharing cannabis & cannabis paraphernalia**		
Sharing before COVID-19 pandemic		
No sharing	107 (46.12)	8 (1.15)
Sometimes	80 (34.48)	324 (46.75)
About half the time	18 (7.76)	160 (23.09)
Most of the time	22 (9.48)	158 (22.80)
Always	5 (2.16)	43 (6.20)
Sharing during COVID-19 pandemic		
No sharing	243 (100.00)	--
Sometimes	--	380 (51.91)
About half the time	--	213 (29.10)
Most of the time	--	109 (14.89)
Always	--	30 (4.10)
**Frequency of non-medical cannabis use**		
Frequency before COVID-19 pandemic		
No use	6 (2.47)	10 (1.37)
Once or twice	33 (13.58)	164 (22.40)
Monthly	62 (25.51)	173 (23.63)
Weekly	63 (25.93)	249 (34.02)
Daily or almost daily	79 (32.51)	136 (18.58)
Frequency during COVID-19 pandemic		
No use	--	--
Once or twice	26 (10.70)	126 (17.21)
Monthly	47 (19.34)	186 (25.82)
Weekly	65 (26.75)	271 (37.02)
Daily or almost daily	105 (43.21)	146 (19.95)
**Mode of non-medical cannabis use** ^b^		
Most reported mode of use before COVID-19 pandemic		
Inhalation	220 (92.83)	661 (91.55)
Non-inhalation	7 (2.95)	31 (4.29)
Most reported mode of use during COVID-19 pandemic		
Inhalation	219 (90.12)	676 (92.35)
Non-inhalation	9 (3.70)	25 (3.42)

a May not add to 100% because of missing data

b Inhalation= Smoking (joint/blunt/bong/pipe), vaporizing plant, vaping oil/concentrates, wax/dab; non-inhalation= Edibles, other oral products (example: pill, tincture, beverage);

**Table 3:** Frequency distribution of policy, state characteristics, and COVID-19 cases by cannabis sharing among a national sample of those reporting non-medical cannabis use, August 2020 – September 2020

	No Sharing	Any Sharing
	n (%)	n (%)
**Policy Level Factors**		
COVID-19 Policy (Median [IQR])		
Average Jun-Aug	16.3 (13.7 – 22.7)	15.3 (11.3 – 19.0)
June	13 (9–16)	10 (8–15)
July	18 (16–26)	17 (12–21)
August	19 (17–26)	18 (13–21)
State’s Legality		
Legal for adult use (non-medical)	146 (60.08)	332 (45.36)
Legal for medical use only	59 (24.28)	216 (29.51)
Legal for CBD use only	35 (14.40)	177 (24.18)
Illegal for all forms of use	3 (1.23)	7 (0.96)
**State Characteristics**		
State Age Distribution (median [IQR])	38.0 (37.00 – 39.60)	38.9 (37.00 – 39.60)
State Percent Urban (median [IQR])	87.87 (81.03 – 94.95)	87.20 (75.07 – 91.97)
COVID-19 Prevalence per 100,000 (median [IQR])	240.96 (240.96 – 447.78)	257.33 (236.17 – 542.59)

Abbreviations. IQR= interquartile range; CBD= Cannabidiol

**Table 4.** Unadjusted and adjusted odds ratios (95% confidence intervals) for cannabis sharing during the COVID-19 pandemic among a national sample of those reporting cannabis use in the United States, August 2020 – September 2020

	Cannabis Sharing	
Primary Analysis	n	OR (95% CI)
Average COVID-19 Policy Score (June to August)		
^a^ Model 1	975	0.72 (0.63, 0.83)
^b^ Model 2	975	0.75 (0.61, 0.93)
^c^ Model 3	960	0.78 (0.62, 0.98)
^d^ Model 4	960	0.78 (0.58, 1.04)

**Sensitivity Analysis**		
COVID-19 Policy June		
^a^ Model 1	975	0.71 (0.60, 0.83)
^b^ Model 2	975	0.75 (0.59, 0.95)
^c^ Model 3	960	0.78 (0.61, 1.00)
^d^ Model 4	960	0.78 (0.58, 1.07)
COVID-19 Policy July		
^a^ Model 1	975	0.76 (0.68, 0.86)
^b^ Model 2	975	0.80 (0.67, 0.95)
^c^ Model 3	960	0.82 (0.67. 0.99)
^d^ Model 4	960	0.82 (0.63, 1.07)
COVID-19 Policy August		
^a^ Model 1	975	0.75 (0.67, 0.85)
^b^ Model 2	975	0.80 (0.67, 0.96)
^c^ Model 3	960	0.82 (0.67, 1.00)
^d^ Model 4	960	0.82 (0.66, 1.02)

Abbreviations. OR= odds ratio; CI= confidence interval

Logistic regression models of sharing prepared cannabis and cannabis-related paraphernalia during the COVID-19 pandemic; modeling odds of any sharing to no sharing per every 5-unit increase in state-level COVID-19 policy.

a Model 1: Unadjusted logistic regression

b Model 2: Adjusted model controlling for state cannabis legality status (legal for adult use (nonmedical), legal for medical use only, illegal [CBD only or fully illegal]), state-level percent urbanicity (continuous), state-level age distributions (continuous), and state-level COVID-19 infection prevalence (continuous)

c Model 3: Adjusted model 2 plus adjustment for individual age (continuous; recentered at mean age and rescaled to 10-year increases), sex (male, female), race/ethnicity (Hispanic/Latinx, non-Hispanic White, non-Hispanic Black, non-Hispanic Other), and education (high school/less than high school, greater than high school)

d Model 4: Adjusted model 3 with robust standard errors for clustered state data

## Data Availability

The dataset with individual-level data used and/or analyzed during the current study are available from the corresponding author on reasonable request. Kaiser Family Foundation’s State COVID-19 Data and Policy Actions and Johns Hopkins University and Medicine COVID-19 Dashboard by the Center for Systems Science and Engineering are publicly accessible through GitHub. U.S. Census data is publicly accessible through the U.S. Census Bureau.

## Abbreviations

COVID-19: Coronavirus disease 2019
CDC: United States Centers for Disease Control and Prevention
CBD: Cannabidiol
IP: Internet protocol
KFF: Kaiser Family Foundation
SD: Standard deviation
IQR: Interquartile range
CI: Confidence interval
